# *Phronesis* in Medical Ethics: Courage and Motivation to Keep on the Track of Rightness in Decision-Making

**DOI:** 10.1007/s10728-020-00398-7

**Published:** 2020-04-30

**Authors:** Aisha Malik, Mervyn Conroy, Chris Turner

**Affiliations:** 1 Health Services Management Centre, University of Birmingham, Birmingham, UK; 2 Emergency Medicine, University Hospital Coventry and Warwickshire, Coventry, UK

**Keywords:** Phronesis, Practical wisdom, Medical ethics, Virtue ethics, Motivation, Decision-making

## Abstract

Ethical decision making in medicine has recently seen calls to move towards less prescriptive- based approaches that consider the particularities of each case. The main alternative call from the literature is for better understanding of *phronesis* (practical wisdom) concepts applied to decision making. A well-cited *phronesis*-based approach is Kaldjian’s five-stage theoretical framework: goals, concrete circumstances, virtues, deliberation and motivation to act. We build on Kaldjian’s theory after using his framework to analyse data collected from a three-year empirical study of *phronesis* and the medical community. The data are a set of narratives collected in response to asking a medical community (131 doctors at various stages of their careers) what making ethically wise decisions means to them. We found that Kaldjian’s five concepts are present in the accounts to some extent but that one of the elements, motivation, is constructed as playing a different, though still crucial role. Rather than being an end-stage of the process as Kaldjian’s framework suggests, motivation was constructed as initiating the process and maintaining the momentum of taking a *phronesis-*based approach. The implications for medical ethics decision-making education are significant as motivation itself is a highly complex concept. We therefore theorise that motivation is required for leading in, continuing and completing the actions of the ethical decision taken. Appreciating the central importance of motivation through the whole of Kaldjian’s framework has implications for cultivating the virtues of *phronesis* and courage to take the right course of action.

## Introduction

Professionalism in medical practice is sought by all physicians. Teaching professionalism is a vital component in any medical curriculum. One key aspect of professionalism is the “defining characteristic” of *phronesis* (practical wisdom) which is acquired when transitioning to professionalism [16: 1]. Dowie argues for introducing practical wisdom in the early formative years of medical students’ ethical development [11]. But what does it mean to cultivate *phronesis* in the early development of a professional and can that cultivation process be described in a way that supports both education and medical practice? Furthermore, what motivates one to engage in the process of wise decision-making? Is it the resistance offered by some to the many forces acting on the decision-maker, such as those underpinned by the calculative thinking that reduce everything into quantifiable measures, losing the person during the process? [14].

Existing notions of the phronesis process have been limited by a lack of empirical study, [21] and there are calls for empirical research into practical wisdom in medical practice [4]. Therefore, based on the results of an Arts and Humanities Research Council (AHRC) funded three-year empirical study we present an advanced theoretical understanding of the *phronesis* process. Primarily this suggests a different conception of how the motivation element in Kaldjian’s [17] framework functions. This has implications for both the educational approach to and the practical application of *phronesis*. First, we explore the notion of *phronesis* and second, we explain the methodology of the empirical study and present the empirical findings and discuss the motivation component of *phronesis*. The theoretical notions built on by this study, in particular Kaldjian’s medical-phronesis framework, are discussed and we propose a rework of the phronesis process to include motivation as pervasive component. Finally, the implications for medical education and practice are outlined.

### Phronesis

Aristotle conceived *phronesis* as the practical wisdom that guides us to achieve the good end (or *telos*) of human flourishing, using the right means [3]. That is, this *telos* ought to be the goal to which one is guided by *phronesis.* Pellegrino considers “*phronesis* as the capacity for deliberation, judgement and discernment in difficult moral situations”, [27: 382] and so, “*phronesis* occupies a special place” and the good doctor is one who can make practically wise judgements [28: 84].

Treatment decisions are fraught with uncertainty and often, medical facts or scientific evidence alone are insufficient, because, to make treatment plans, a physician requires not just clinical knowledge but also an understanding of patients’ social circumstances, patients’ (or carers’) values / beliefs and available resources.

Kaldjian argues that the best model for making clinical decisions is not *purely* scientific judgement but Aristotle’s model of *phronesis,* where scientific, socio-cultural and ethical knowledge are integrated [17]. As an intellectual virtue, *phronesis* plays an “integrative role which helps one [or a peer group] to act virtuously in an overall way” [22: 303] *Phronesis* involves the ability to know and deliberate about ends and goals that are worth pursuing and the means (right means) most likely to accomplish those goals [20].

A large corpus of literature has started to build around *phronesis* in medicine but is mainly theoretical or philosophical. Aristotle’s original writings on *phronesis* are interpreted by Dunne who suggests that, “we are accustomed to looking at *phronesis* as knowledge that will guide action; but that *phronesis*…also arises from good action” [12: 290]. In other words it arises from habit: being *good* and doing *good* because it is *good*. Theoretical conceptions of *phronesis* including Aristotle’s have been explored in detail by Kristjannson [22], who argues that *phronesis* is just a putative ideal with differing accounts of the concepts in the literature.

Attempts to study clinical-judgement as *phronesis* using empirical data are rare [21]. In this paper we present an inductively driven grounding of the virtue of *phronesis* inferred from the experiences of a wide range of medical practitioners and explore them against the background of Kaldjian’s *phronesis-*based framework [17, 18]. Kaldjian’s framework has five core elements [17: 559, 561] as shown in Table 1.

**Table 1:** Kaldjian’s medical-*phronesis* framework

Core element 1	Pursuit of worthwhile ends (goals) derived from a concept of human flourishing
Core element 2	Accurate perception of concrete circumstances detailing the specific practical situation at hand
Core element 3	Commitment to moral principles and virtues that provide a general normative framework
Core element 4	Deliberation that integrates ends (goals), concrete circumstances, and moral principles and virtues
Core element 5	Motivation to act in order to achieve the conclusions reached by such deliberation

According to Kaldjian these five elements are useful, necessary, and sufficient constituents of a wise decision-making process, and “form a trajectory of moral decision- making” [18: 227]. Although the first three elements, worthwhile goals, concrete circumstances and commitment to moral principles and virtues, on a MacIntyrean [23] reading would be a part of ongoing inter and intra-practice moral debate, and so may be considered as occurring simultaneously in the debates, the fourth element of deliberation to integrate and reach a decision, can only really come in to play once the first three have been considered. Following this deliberative process, Kaldjian’s framework provides the last element: “5. Motivation to act in order to *achieve the conclusions reached by such deliberation*” [17: 559, emphasis added]. It seems that motivation only comes in as a final stage in the Kaldjian framework—as a ‘motivation to act’ stage.

When analysing interviewees’ narratives, we found that collectively our participants implicitly engage with Kaldjian’s core elements when making treatment plans; they are not alien to the medical community. We have termed this phenomenon ‘*Phronesis*-in-action’. However, our findings suggest that motivation plays an essential, albeit a slightly different, additional, role: rather than being a single stage of the process it continuously drives the process of *phronesis,* maintaining the momentum.

We now explain the methodology used to gather data and present the findings. We present narratives as illustrations of the contextual moral reasoning employed and the core elements engaged with (or not). We then discuss phronesis-in-action and the role of motivation.

## Methodology

The aim was to understand the meaning of ethical decision-making and the virtue ethics of interviewees’ practice. Narratives offer a way to access the meanings to participants in the form of virtues they are grappling with in their practice [5]. The study grows from three distinct and compatible ontological roots [6]. First, we draw from humanities and the neo-Aristotelian virtue ethics philosophy of MacIntyre [23] who suggests that narrative operates as a grounding for passing on the ethics of any practice to others in the same or related circumstances. He further argues that human action is only understandable when the action is related to the intentions (purpose) and contexts (settings) with the ordering creating a narrative. Czarniawska [8] argues that narratives play a fundamental part in socially constructing subjective reality, which we argue is crucial to any methodology designed to study *phronesis*. Second, we draw on Flyvbjerg et al.’s [13] ethnography of *phronesis* to support the context and settings of the narratives for a film production element of the study. Finally then is the third root based on a participatory video approach from Schugurensky [30] where participants and the research team are involved in iteratively producing a series of film episodes that convey in the acting a consolidated set of 15 virtues, including *phronesis* based on the narratives from the interviewees [7].

### Methods

#### Ethics

Participants were sent the information sheet 48 h before the interview and informed of the right to withdraw.

Ethics approval for this study was obtained from the research ethics committee at the Universities of Birmingham, Nottingham and Warwick, and the Health Research Authority.^1^

#### Data Collection

Our participants included medical student and experienced doctors and were a mixed cohort of gender and specialisations. Participants were informed in the information sheet of our interest in hearing their stories of wise (or unwise) decisions made.

Narrative interviews were conducted to collect primary data and consent obtained a priori. We started by asking our participants what it means to them to make ethical decisions for their patients, and the different issues they take in to account before making a final decision. We specifically sought anonymized examples of decisions made and explored them in depth. Most of the interviews were conducted face to face; few were via telephone. Although there was an *aide memoire*, it was rarely resorted to. Ethnographic observations were conducted to contextualize the narratives.

#### Analysis

The nature of *phronesis* was analysed using two theoretical lenses: MacIntyre’s practice virtue ethics [23] and Kaldjian’s [17] core elements (see Table 1) for medical *phronesis*. The findings of the former practice virtue ethics theoretical lens analysis are presented elsewhere [7]. Here we focus on the analysis and findings from using Kaldjian’s medical-*phronesis* framework. Our interviewees’ stories provided first hand experiences to compare with Kaldjian’s concept of *phronesis* and how it is socially constructed in the moral development of medical practice [21]. 131 participants (medical students and practicing doctors) were interviewed for the original research, however, we only included practicing doctors’ narratives (foundation years^2^ and those with more than 5 years’ experience) (Tables 2, 3) for analysis through Kaldjian’s framework.

**Table 2:** Interviewees’ narratives exhibiting core elements of medical-phronesis framework

Code	Goals/outcomes (1)	Concrete circumstances/context (2)	Virtues/principles/normative framework(3)	Integrating 1, 2 and 3	Motivation to initiate the process and implement the decision reached
BX01	**+**	**+**	**+**	**+**	**+**
BX02	**+**	**+**	**+**		
BX03	**+**	**+**	**+**		
BX04	**+**	**+**	**+**	**+**	**+**
BX05	**+**	**+**	**+**	**+**	**+**
BX06	**+**	**+**	**+**		
BX07	**+**	**+**	**+**	**+**	**+**
BX08	**+**	**+**	**+**		
BX09	**+**	**+**	**+**		
BX10	**+**	**+**	**+**		
BX11	**+**	**+**	**+**		
BX12	**+**	**+**	**+**	**+**	**+**
BX13	**+**	**+**	**+**	**+**	**+**
NX01	**+**		**+**	**+**	**+**
NX02	**+**	**+**	**+**	**+**	**+**
NX03	**+**	**+**	**+**	**+**	**+**
NX04	**+**	**+**			
NX05	**+**	**+**			
NX06	**+**	**+**			
NX07	**+**	**+**			
NX08	**+**	**+**	**+**	**+**	**+**
WX01	**+**				
WX02	**+**				
WX03	**+**				
WX04	**+**	**+**	**+**	**+**	**+**
WX05	**+**	**+**	**+**	**+**	**+**
WX06	**+**	**+**	**+**	**+**	**+**
WX07	**+**				
WX09	**+**				
WX10	**+**	**+**	**+**	**+**	**+**
WX11	**+**

**Table 3:** Interviewees’ narratives exhibiting core elements of medical-phronesis framework

Code	Goals/outcomes (1)	Concrete circumstances/context (2)	Virtues/principles/normative framework (3)	Integrating 1, 2 and 3	Motivation to initiate the process and implement the decision reached
W101	**+**	**+**	**+**	**+**	**+**
W101-FP	**+**	**+**	**+**	**+**	**+**
W102	**+**	**+**	**+**		
W103	**+**	**+**	**+**		
W104	**+**	**+**	**+**		
W104 -FP	**+**	**+**	**+**		
W105	**+**	**+**	**+**		
W106	**+**	**+**	**+**		
W107	**+**	**+**	**+**		
W107 -FP	**+**	**+**	**+**		
W108	**+**	**+**	**+**		
W108-FP	**+**	**+**	**+**	**+**	**+**
WFY2-01	**+**	**+**	**+**		
WFY2-02	**+**	**+**	**+**		
WFY2-03	**+**	**+**	**+**		
WFY2-04	**+**	**+**	**+**		
WFY2-05	**+**	**+**	**+**	**+**	
WFY2-06	**+**	**+**	**+**	**+**	**+**
N101	**+**	**+**		**+**	**+**
N102	**+**	**+**	**+**	**+**	**+**
N103	**+**	**+**	**+**	**+**	
N104	**+**	**+**	**+**	**+**	
N105	**+**	**+**		**+**	
N106	**+**	**+**	**+**	**+**	
N107	**+**	**+**	**+**	**+**	
N108	**+**	**+**	**+**	**+**	
B101	**+**	**+**	**+**	**+**	
B102	**+**	**+**		**+**	
B103	**+**	**+**		**+**	
B104	**+**				
B104-FP	**+**	**+**	**+**	**+**	**+**
B105	**+**	**+**		**+**	
B105-FP	**+**	**+**		**+**	
B106	**+**	**+**	**+**	**+**	**+**
B107	**+**	**+**	**+**	**+**	
B108	**+**	**+**	**+**	**+**	
B108-FP	**+**	**+**		**+**	
B109	**+**	**+**			
B109-FP	**+**	**+**			
B110	**+**	**+**	**+**	**+**	
B110-FP	**+**	**+**	**+**	**+**	**+**
B111	**+**	**+**	**+**	**+**	**+**
B111-FP	**+**	**+**	**+**	**+**	**+**
B112	**+**	**+**	**+**	**+**	**+**
N101-F	**+**	**+**	**+**	**+**	**+**
N105-F	**+**	**+**	**+**	**+**	**+**
N107-F	**+**	**+**	**+**	**+**	**+**
NFY2-01 (2)	**+**	**+**	**+**	**+**	**+**

Using Kaldjian’s framework is important for two reasons. First, there is a call to empirically validate whether the various ethical decision-making frameworks are fit for purpose [25]. Second, since the present research is on *phronesis*, a *phronesis*-based framework, which Kaldjian provides, is needed for developing theory (regarding ethical decision-making) from the findings.

## Findings

The stories were about interviewees’ practice or the practice of others whom they work with, and whether they perceived them to be good/wise or not so good/ unwise decisions. In order to provide an insight into our interviewees’ understanding of wise as opposed to unwise decisions, as judged by the interviewees themselves, we present the findings that relate to each of the five core elements (Table 1) and offer a different perspective on motivation.

### Goals of Care to Pursue

The goals gleaned from the narratives of our interviewees were mainly that patients’ wishes are respected: treat and improve their health or preferring quality of life rather than quantity. However, there were instances when patients’ perspective “*is different to our perspective as a professional*” (BX12). Similarly, WX04 narrated how a patient admitted to the ward was not happy with the decision that doctors had made for him. The decision was to book the patient “*for an endoscopy to change the PEG (Percutaneous endoscopic gastrostomy) [tube]*” (WX04) as was requested by the care home. However, WX04 realized:

[T]hat he (patient) was not willing to have the feeding and he was willing to stop his feeding – he didn’t want the PEG reinserted again (WX04).

WX04 acknowledged that by virtue of having capacity, the patient had the right to have the tube *“removed…and sent him back to the care home, [to] have food and drink as much as he can, so eating as much as he can and if he got any infection or any other problem, let him go in peace as he [patient] wanted… *(WX04).

Another interviewee (BX05) narrated how a brain-injured patient was on medication that “*knocked him out”*. This clinical goal, thought necessary by the cardiologist, was not what this patient’s family or BX05 wanted. It was observed that medical and social goal-oriented discussions were taking place between the team looking after this patient—for them maintaining this patient’s functionality was an important goal:

Keeping the patient’s brain perfused so that he is able to function; although it may shorten life there is some quality of life” and for that it was necessary to discontinue “all the medication that might knock him off centrally, making him drowsy. I’ve got to do everything I can both medically and, from the therapy perspective, to optimise his function because that will tell us how far he can rehabilitate and where he’s going to go to after hospital (BX05).

Another interviewee narrated, in relation to another patient, that the goal of medicine should be to realize when further intervention is futile, in fact it is harming the patient:

[I]t was inappropriate to keep putting her (patient) through tests that [made] her uncomfortable…. what are we really achieving, and so, I went out and I spoke to the Registrar. He was like, “Yeah, I don’t think we should do anything for her.” Like, with the discussion with the daughter, and the grandson. I think that was the right thing to do [not to intervene] (W101-FP).

There are instances when the discordance alluded to above between the goals of care as viewed by doctors and patients, or their family, are irreconcilable and unwise decisions are made, adversely affecting the outcome. Narrating an incidence, this interviewee said:

Recently a patient was offered an operation by a surgeon that was clinically the right operation to be offered, but the way it was communicated to the patient, they have refused to have the operation. They think it’s completely not the right thing for them… (NX03).

### Perception of Concrete Circumstances

Most interviewees considered that accurate perception of concrete circumstances detailing the specific practical situation at hand are important in making decisions. These circumstances would be clinical facts, social circumstances and the context in which the healthcare is delivered. Sometimes clinical facts may lead doctors to have a narrow focus of action. For instance, WX04 narrated that although he had been informed by another doctor that the patient was booked for endoscopy—“*to change the PEG”*—because that is what the care home requested, WX04 engaged with the patient and found:

He was – sometimes just saying ‘no’ with the head and things, so we came to the point that through our conversation, I realised that the patient has capacity, perfect capacity when you take the time to allow him to express and I acknowledged… he did not want the tube reinserted (WX04).

Others were aware of the need to consider concrete circumstances based on guidance:

So, within the WHO ICF classification there’s a very clear definition of disability- so the pathology, impairment, activity limitation, participation limitation and then contextual factors- physical structures around the person, like their caregivers etc. and the legal, contextual factors… (BX05).

### Commitment to Moral Virtues and Principles

What informed these interviewees to make decisions that were for the good of the patient (in patient’s best interest), was commitment to professionally driven virtues and principles. In this regard, the following virtues were gleaned from the interviewees’ narratives: respecting patient’s values, interpersonal communication, a balanced holistic approach, recognising limits to treatment, seeking advice and courage.

#### Respecting Patient’s Values

Most interviewees considered respecting patients’ values and beliefs as important:

A huge part in my decision making is influenced by I think the patient’s values and beliefs, and the family’s values and beliefs as well (BX01).

Being farsighted and spending time to try and understand what is it that the patient really wants is helpful:

So, I discussed with him and I really get to that information; it took me obviously more than ten minutes… [and because] that was his (patient’s) decision, with capacity, after talking to him…. I got to what the patient really wanted to happen; I respect that, and I help him in the best way possible (WX04).

#### Interpersonal Communication

Some interviewees narrated experiences where the doctor was unable to communicate to the patient a procedure (or treatment plan) that was probably the right intervention. Poor interpersonal communication resulted in distrust and the patient refused a beneficial intervention:

…. the way it was communicated to the patient, they have refused to have the operation. That made me think well, this doctor could be fantastic in terms of his clinical acumen but if his communication is not there, well, that’s not going to give a favourable outcome (NX03).

#### Holistic Approach

Interviewees considered that constructing a holistic view of the patient as a person was essential. Patients are not a body that requires readjustment of the biochemical milieu, nor is the patient divided into organs working in isolation, as BX05 reiterated:

[W]e have a holistic view of the whole person, so they’re not just a heart that’s been damaged with the rest of the body attached to it; we’ve got to look at the whole picture… that really is another way of saying the holistic biopsychosocial model (BX05).

A senior doctor, it was observed, sat down with a foundation year doctor, at her suggestion, to discuss another older patient with complex health and social problems.

#### Recognizing Limits of Treatment

W101-FP narrated how continuing to treat a patient was not “*achieving*” anything; actually, it was harming the patient. Another narrated a similar experience:

For me, it's about the cost of suffering, prolonging a life where… And we get questioned a lot …..Well, we can see the situation's futile, why don't we withdraw sooner? (BX12).

While another interviewee praised how a consultant they worked with “*seem to be very good at seeing problems, seeing multiple solutions, and making decisions and plans that work in different situations*” and so made prompt alternate treatment plans:

So, [the] consultant will go on a ward round say, ‘I think we should do X, but if when we do X this happens, do Y, and if that doesn’t work then we’ll do Z.’ And I feel like they are wise and thought out decisions. So, I think maybe they can see the outcome of the decision that they make in the future, and how that relates to everything else (BX01).

#### Seeking Guidance

There were those who were of the view that discussing with senior doctors and /or peers helps to achieve a good end:

He [registrar] reviewed the patient, and we got the Medical Registrar as well, who came in and saw her, and he was, kind of, the most senior medical person there at that point, ‘cause it was during the night, and he made the decision. He was like, “Yeah, I don’t think we should do anything for her.” I think that was the right thing to do (W101-FP).

It was also observed that mutual support and compassion were on display in circumstances where a lot of rapid decisions were being made.

#### Courage

Narrating an incident where an emergency medicine Consultant made a decision that challenged the decision of other doctors regarding a post-cardiac arrest patient, WX09 was of the view “*that emergency medicine consultant actually did make a wise ethical decision for that patient by playing “What if -?”* and though WX09 was not initially in agreement with this consultant’s decision admired the consultant’s courage to do what was right for the patient:

So, for a good ethical decision, that emergency medicine Consultant absolutely challenged how I viewed that patient and I would like to think it’s probably changed how I view other patients in the future (WX09).

### Deliberation to Integrate Concrete Circumstances, Moral Principle/Virtues to Achieve the ‘Good End’

An important element in reaching a wise decision is integrating the particular circumstances of the case with the best interest of the patient whilst being guided by principles or virtues. This, though challenging, results in a good decision:

So what I need to do is try and optimise his [patient’s] health in general to enable his brain to function as well as it possibly can… Then look at modifying the factors around him, so looking at whether it’s too noisy, whether he gets communication in the right way, all those sorts of things. But the essential thing is getting him as fit as possible (BX05).

To make good ethical decisions some flexibility is required, otherwise:

If you have already in your mind motivated to do a particular thing, then the conversation probably becomes biased, so sometimes you just go with the open mind, and then with the conversation with the patient, you think what would be the next step (BX04).

W101FP had the foresight to predict the course of a decision taken and integrating all aspects decided:

[T]o make him [Registrar] see that side of it, and he (Registrar) agreed. He (Registrar) reviewed the patient… [The decision made should have] “been made as soon as they came in to hospital; of not to do any more, and to make them comfortable. And then as soon as that decision was made, everything just became a lot calmer, and the family were happier, the patient was happier. It was just a shame that it took, kind of, the whole day for that to happen (W101FP).

This then makes one realize that a good medical decision and an ethical decision are intertwined:

Was it a good ethical decision or was it just good medicine?… is a bit of a challenge but it probably became good medicine because it was a good ethical decision (WX09).

### Motivation (to Initiate the Process and to Act to Achieve the Conclusions Reached by the Process of Deliberation)

Interviewees seemed to be constructing motivation at all stages of the process which suggests that they felt this was an important and pervasive part of a *phronesis* -based approach. Motivators mentioned include best interest of the patient, goodwill, avoiding harm, respecting the patient’s (or the family’s) autonomous decision and engage with the process to act on the decision made.

For example, looking at motivation and goals. In circumstances such as those narrated above where the clinical goals clashed with the goals considered important by the patient (or the family) many interviewees, motivated to act in the patient’s best interest, engaged with the *phronetic* process (see Tables 2, 3)

There are times when the goals to do what is good for the patient and the goals of the organization are divergent. WX04 narrated how he, in the face of disagreements with the organization and colleagues, was motivated to decide in his patient’s best interest:

So, as a decision-maker we are pushed [in] different ways … our organisations pulling from us, the patient, the family, we have our own knowledge…. they are pulling in different directions. Sometimes they are all in the same direction which is good and that makes it easy in these cases, but sometimes they are pulling in different directions…I go for what was best for the patient and [in] agreement with the patient (WX04).

Motivation is constructed at the outset of the ethical decision-making process:

*“You need to have the desire, the motivation, to do a thing, then you're led to make that decision” (BX04).* The rationale for not delaying and being motivated to act is included in this further excerpt:

Lots of factors that come into play. It’s what the situation is, what is the state of the patient, where if I don’t make a decision, what will happen to the patient, basically? What will be the consequence of the decision that I will be making? If I don’t make a decision, then if I delay the situation, what would happen to the patient? (BX04).

The motivational force of “*doing good*” and “*best interest*” holds traction with others too e.g. participants in our follow-on project stated that:

You have to be motivated in the first place to try to get to the right decision…. then motivation comes in recurrently [throughout] the process, because what you think is the best decision in the circumstances, that may not be what the organisational best decision might be- so all they really care about is hitting a target, they kind of don’t care how you get there. So, there are pressures on staff to perform and to make certain decisions, which are not necessarily congruent with good patient care” (Int3-02). Furthermore: “It is essential to do what is in the best interest of the patient and for this it helps to be able to understand your values, so you are motivated to do the right thing for the patients (Int3-02).

In these circumstances, some doctors were of the view that one must be motivated at the outset to engage with the patient and have the courage to act in the best interest of the patient.

BX05, motivated by the best interest of the patient, thinks in terms of the biopsychosocial model [32] wherein interaction between biological, psychological, and social factors calls for a holistic decision: BX05 realized that although the cardiologist’s medication will improve the patient’s long-term outcome from a cardiac perspective, the patient’s functionality was markedly compromised, which prompted BX05 to say to the cardiologist (who was also looking after the same patient):

Actually, we’re stopping some of your medication. We accept it may, on average, result in a shorter lifespan but if it’s something that’s going to keep his blood pressure at a level that keeps him cerebrally perfused and able to function then he’s got quality (BX05).

Other interviewees also narrated that “*for as long as [they’ve] known the health service there is a huge level of goodwill*”(BX10) and “*enthusiasm*” (NX02) where the primary motivation is “*constantly thinking about what is in the patient’s best interest”* (BX05) otherwise as a clinician *“all you want to do is exciting procedures*” (NX08).

In order to make decisions that take into account the many circumstances around the patient their motivation also comes through even to the point of moving away from clinical guidelines:

So they’re not just a heart that’s been damaged with the rest of the body attached to it; we’ve got to look at the whole picture and the cardiologists I’ve had debates with have always been very happy to take on board that holistic perspective and see the limitations of their treatment. And have not had a problem in going off protocol when there’s clearly a best interest’s issue (BX05).

## Discussion

### Phronesis-in-Action: Motivation Drives the Process and Virtues are Integral

Our interviewees’ actions are “based on an engaged, embodied and enacted judgement that links knowledge, experience and virtue”, and a dialectical relationship between patient’s desires, circumstances and virtues fostering a morally right action [1: 244]. Using Kaldjian’s core element framework is beneficial [4, 10] and though, sometimes tension exists between a clinical concept of health (focussing on eliminating the physiologically abnormal state) and a well-being concept (focussing on the subjective experience of the patient) [19] these can be mitigated by using Kaldjian’s framework.

Integrating goals, circumstances and ethical values is necessary to achieve the larger purpose of the patient’s good [17], spurred on by motivation. Motivation initiates the process and maintains the momentum. Motivated to do good for the patient, free of the “dominance of calculative thinking” [14], urged the doctors to ask, “What do I want?” before answering “What should I do”? [24: 4]. That is, what is in *this* patient’s best interest and then, initiate the *phronetic* process.

**Fig. 1:**
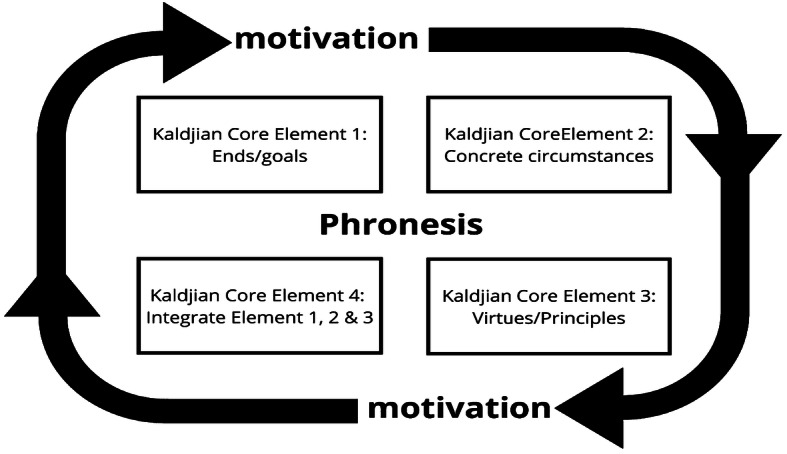
‘Phronesis-in-action’-Kaldjian Core Elements Framework

Our findings informed the ‘*Phronesis*-in-action’—Kaldjian core elements framework (PIA-Kaldjian framework) (Fig. 1). By that, we mean an inductively informed framework that helps doctors make wise decisions in their clinical practice. While in Kaldjian’s medical-*phronesis* framework (Table 1) the five elements form a trajectory of moral decision making [18], with motivation at the conclusion of the process- “to achieve the conclusions reached by such deliberation” [17], in our PIA-Kaldjian framework motivation is continually present. Decision-makers’ motivational rationality, consistent with their moral identity or reasoned emotions [9] are “expressed both in action and in those states of mind that motivate [one] to act” [24: 6]. We argue, based on the PIA-Kaldjian framework, that the *phronetic* process does not take place in a “motivational void”; our interviewees had “motivational tendencies” to initiate the process; it is not subsequent to practical reasoning [2: 29]. Aristotle’s work on virtue ethics for the individual can be interpreted as supporting the argument of motivation being a separate, pervasive part of the application of *phronesis* on any particular ethical issue being addressed [3]. That is, motivation drives and sustains the process of what is the right thing to do and implement it, under the circumstances. Specifically, the interviewees were motivated to initiate and maintain the *phronetic* decision-making process. This empirically-evidenced argument is supported by the call for a revised empirically- based account of *phronesis* development and the theoretical argument put forward by Darnell et al. in the third of their four component model which is “…that of possessing a blueprint of the good life that enables individuals to adjust their own moral identity to accord with the blueprint, thereby furnishing it with motivational force” [9: 122] This moral identity adjustment infers the acquisition of consistent motivation throughout for *phronimoi* or those wise peer group practitioners who want good for their patient and their communities. This notion comes across in the narratives or ‘narrative identities’ [29] we have included here.

The motivation to act in the best interest of the patient is seen in clinical encounters reported by others. For instance, David Sokol, in a recent BMJ article writes of an incident where a meeting was arranged to diffuse tensions between doctors and carers of a patient in critical care. Sokol points out that “we were all motivated by the desire to do what was in the best interest of the patient”, which was greeted by “nods of agreement” [31]. One could argue that this role of motivation and virtues is a given since it entails “a desire to make actions consistent with beliefs” [18: 227], as an intention-consequence complex. It is, however, important to acknowledge the pervasive role of motivation and virtues in ethical decision-making as a motivation-consequence complex.

### Implications for Ethics Education

According to Dunne [12], who draws on Aristotle, *phronesis* represents the achievement of practice excellence and evolves alongside technical and theoretical knowledge. So that the ‘medical-technical discourse’ is transformed to practical wisdom in practice [4], medical *phronesis* requires attention and training, in both medical education and the workplace [22]. Introducing phronesis in the formative years of ethical development is important [11]. For this, it is essential to devise a contextual rather than just a theoretical ethics curriculum [15]. We argue that Kaldjian’s core elements are mirrored in many of our interviewees’ narratives. However, because motivation is more of a constant and continuous driving concept (Fig. 1) we suggest a discussion on the motivation to make ethical decisions is vital before progressing to a detailed discussion on Kaldjian’s framework. We also suggest a discussion regarding the courage to maintain motivation when a medical practitioner is on a contentious pathway of decision-making. Dinoff [10], who carried out a Kaldjian-based study, was highly supportive of the framework and uses a quote from William Penn: ‘*Right is right, even if everyone is against it, and wrong is wrong, even if everyone is for it*’ which emphasises the importance and relatedness of another one of the virtues which our participants mirrored-courage.

## Conclusion

The five core elements of Kaldjian’s framework form a trajectory for implementing a theory of *phronesis:* goals, concrete circumstances, virtues, deliberation and motivation to act. However, we suggest that motivation plays a different role to just being the last stage in the process. Motivation is pervasive: it initiates the process and maintains the momentum throughout urging the doctor to integrate the practical elements (goals and concrete circumstances) and the guiding principles (virtues) to achieve the ‘good’/right end.

We therefore argue that the modified ‘*Phronesis*-in-Action’-Kaldjian framework (Fig. 1) is useful when making decisions, bedside teaching, medical ethics education and in debriefing sessions. This is because a discussion on practitioners’ motivational rationality to pursue an ethical (*phronetic*) approach must be determined (and maintained) for each participant to ensure their continued allegiance to the decision- making process that is in the best interest of their patient. The courage to pursue what they believe to be the right way to go about ethical decision-making is also intrinsic to this process.

### Future Research

Our project was in a different medical culture—different insofar as the NHS is free at the point of delivery—than the US system where Kaldjian framework has so far been evaluated (medical services are charged to the patient) and has implications for future research. Our research findings have provided some grounding for Kaldjian’s framework being applicable universally. Future research in other medical practice communities would help validate (or invalidate) its application in different contexts. It will also be important to see whether the motivational aspect that we found is the same, and is pervasive, in medical practice, globally.

### Limitations

Although the findings are inductively derived from the largest number of doctors to date [6], they may not be extrapolated to all doctors. Nevertheless, considering that it is a specific practice community being studied, and that “physicians appear… to be part of a global medical practice with a shared medical culture” [26], we think that this work may be applicable to other medical practices, depending on particulars and to be used with practical wisdom (*phronesis*) to determine where on the virtue continuum a decision and action based on that decision sits. In this respect we offer this as a working hypothesis to be tested both by the academic and practitioner community, globally.

## Footnotes

1 Ethics approval Letter No: 18/HRA/0203.

2 Foundation Year 1 and 2 doctors are registered doctors who have recently graduated from medical school and are part of a 2-year training programme.
